# Autism care pathway in Europe

**DOI:** 10.1192/j.eurpsy.2023.2435

**Published:** 2023-09-11

**Authors:** Maria A. Mendez, Bethany Oakley, Roberto Canitano, Antonia San José-Cáceres, Michela Tinelli, Martin Knapp, James Cusack, Mara Parellada, Pierre Violland, Jan R. Derk Plas, Ricardo Canal-Bedia, Alvaro Bejarano-Martin, Declan G.M. Murphy, Vinciane Quoidbach, Celso Arango

**Affiliations:** 1 Instituto de Investigación Sanitaria Gregorio Marañón, Hospital General Universitario Gregorio Marañón, Madrid, Spain; 2 CIBERSAM (Mental Health Networking Biomedical Research Centre), Madrid, Spain; 3 Department of Forensic and Neurodevelopmental Sciences, Institute of Psychiatry, Psychology and Neuroscience, King’s College London, London, UK; 4 AIMS-2-TRIALS consortium; 5 Azienda ospedaliero-universitaria Senese, Siena, Italy; 6 Department of Health Policy, London School of Economics and Political Science, London, UK; 7 Autistica, London, UK; 8 Department of Child and Adolescent Psychiatry, Hospital General Universitario Gregorio Marañón, Madrid, Spain; 9 School of Medicine, Universidad Complutense, Madrid, Spain; 10 Universidad de Salamanca, Salamanca, Spain; 11 European Brain Council, Brussels, Belgium

**Keywords:** autism, diagnosis, early intervention, early screening, policy recommendations

## Abstract

**Background:**

Autism is a lifelong complex neurodevelopmental condition that affects brain development and behaviour with significant consequences for everyday life. Despite its personal, familial, and societal impact, Europe-wide harmonised guidelines are still lacking for early detection, diagnosis, and intervention, leading to an overall unsatisfactory autistic person and carer journey.

**Methods:**

The care pathway for autistic children and adolescents was analysed in Italy, Spain and the UK from the perspective of carers (using a survey aimed at caregivers of autistic children 0–18 years old), the autistic community, and professionals in order to identify major barriers (treatment gaps) preventing carers from receiving information, support, and timely screening/diagnosis and intervention.

**Results:**

Across all three countries, analysis of the current care pathway showed: long waits from the time carers raised their first concerns about a child’s development and/or behaviour until screening and confirmed diagnosis; delayed or no access to intervention once a diagnosis was confirmed; limited information about autism and how to access early detection services; and deficient support for families throughout the journey.

**Conclusions:**

These findings call for policy harmonisation in Europe to shorten long wait times for diagnosis and intervention and therefore, improve autistic people and their families’ journey experience and quality of life.

## Introduction

Autism is a lifelong, complex, early onset condition that affects brain development and behaviour, characterised by difficulties in social communication, restricted and repetitive patterns of behaviour, interests, or activities, and sensory issues, which have significant consequences in daily life [1–3]. Its prevalence has steadily increased over the past 30 years [4] with rate estimates in European studies varying between 1 and 2% in school-age children [5–8]. Outcomes in autism are highly variable, and intellectual ability is not the only factor in predicting a better outcome [9]. Early intervention can play a crucial part in social-communicative and emotional development, which acts as a bridge for more complex abilities. The primary goal of early intervention is to maximise functional independence and quality of life [10–14].

Autism has a considerable functional and financial impact on those affected, their families, and society [15, 16]. Repercussions include everything from high health expenditure to low employment prospects, poor mental health, and wellbeing [17–19]. By the same token, a high percentage of carers report giving up or cutting back on work to care for an autistic child [20]. Although the United Nations (UN) and the World Health Organization (WHO) have recognised autism as a public health issue, it has received little attention from European public health services [11, 21]. Despite this great impact, Europe-wide consensus and support for early detection, diagnosis, and intervention is lacking. Furthermore, individual countries may follow local or regional guidelines. All of this leads to an overall unsatisfactory journey for autistic people and their families [20, 22].

In the ideal care pathway, each person suspected of being autistic would have the right to an initial medical evaluation, known as screening, which is usually carried out by a paediatrician. Subsequently, if the overall developmental assessment indicates the need for a more comprehensive assessment specific to autism, a diagnostic interview should follow. This evaluation should be performed by a multidisciplinary team in which all team members have received training in autism, and at least one member must have training in the evaluation and diagnosis of autism using standardised instruments [23]. Unfortunately, families with young autistic children describe this process as complex, long, and stressful [24–26]. Timely screening and early diagnosis are of great importance in order to make an accurate diagnosis, identify individual needs, and guarantee the implementation of an intervention that meets those needs, which should start soon after diagnosis [27].

The aim of a care pathway is to enhance the quality of care by improving patient outcomes, promoting patient safety, increasing patient satisfaction, and optimising the use of resources [28]. With this in mind, in 2016, the European Brain Council (EBC), an organisation promoting research on brain health and disorders in Europe, initiated a study called the Value of Treatment (VoT). In its second round (VoT2), in 2018, the EBC deemed it necessary to add case studies on neurodevelopmental conditions such as autism. The VoT2 project aims to examine the value of early diagnosis and intervention and to assess the benefits of coordinated and multidisciplinary care patterns on patient outcomes and socio-economic impacts resulting from best-practice healthcare interventions, in comparison with current care or no treatment.

In this article, we present findings on the journey of carers through awareness of early signs, diagnosis, and follow-up support. Our overarching objectives are: (1) to identify the current treatment gaps and needs of the autistic population, to help us identify the underlying causes and propose solutions to these gaps; and (2) to propose policy recommendations on how to improve the European care pathway.

## Methods

To assess the care pathway, we formed an autism working group (WG) consisting of members of the European Society for Child and Adolescent Psychiatry (ESCAP), representatives of the Global Alliance of Mental Illness Advocacy Networks-Europe (GAMIAN-Europe), the European Federation of Associations of Families of People with Mental Illness (EUFAMI), members of the autism community (autism representatives part of the AIMS-2-TRIALS network), Autistica, Autism Europe, economic and mental health policy experts from the London School of Economics (LSE), Universidad Complutense de Madrid (UCM), Instituto de Investigacion Sanitaria Hospital Gregorio Marañón Madrid (IISGM), King´s College London (KCL), Universita degli Studi di Siena (UNISI), Universidad de Salamanca (USAL), the Belgian and Spanish Brain Councils, and representatives of the pharmaceutical industry (i.e., Roche and Servier).

The care pathway of autistic children was analysed from the perspectives of carers and professionals in order to identify major barriers (treatment gaps) preventing them from receiving information, support, timely screening, diagnosis, and treatment/intervention. In order to assess this, we conducted a rapid literature review of the current care pathway including a review of existing guidelines in Europe. We also conducted a survey aimed at carers of autistic children ages 0 to 18 living in Italy, Spain, and the UK. Additionally, members of the WG met regularly between 2019 and 2021 to identify the main treatment gaps and causal factors of these gaps, prepare a survey (to evaluate service users’ unmet needs), discuss survey results, and propose policy recommendations. The following critical points of the care pathway were addressed:

- Screening/diagnosis after carers raise first concerns to health professionals.
- Intervention/treatment once the diagnosis is confirmed.
- Information about access to services and support for families and carers of autistic children.

### Survey development

We drafted a survey based on the one conducted by the Autism Spectrum Disorder in the European Union (ASDEU) network [22] with some modifications, including extending the age range to comprise 0 to 18 years. This survey was prepared by M.A.M. in English, reviewed by C.A., D.G.M.M., B.O., R.C., J.C., and members of the autism community, then translated to Spanish by M.A.M. and Italian by R.C., and adapted by B.O. and R.C. to reflect local services and local ethics committee suggestions and regulatory guidelines. To view complete survey please refer to Supplementary material.

The survey was developed using the REDCap platform, a secure, web-based software platform, designed to support the collection and management of data for research studies, managed by the Hospital General Universitario Gregorio Marañón [29].

### Inclusion criteria

The survey was aimed at carers of autistic children ages 0–18 residing in Italy, Spain, and the UK in order to obtain data about their access to and experience with local services. Only participants who signed an online informed consent were able to complete the survey and enrol in the study. The reasons for exclusion were: having an autistic child older than 18, not being a resident in Italy, Spain, or the UK, or not signing the online informed consent.

### Recruitment procedure

Participants were identified from the researchers’ institutions, organisations for people with autism and their families, and autism-related professional organisations. The survey was made available online and disseminated through social networks including Twitter, Facebook, Instagram, LinkedIn, and so on. via the link generated by the REDCap platform. An invitation and summary information about the study was included with this link. People who wanted to participate were directed to the first page of the study where they were provided with more detailed information about the study and were asked to give consent. In order to increase recruitment, frequent reminders were sent to the aforesaid collaborators who kindly sent reminders or re-posted invitations on their social platforms.

### Ethical approval

Ethical approval was given by the Ethics Committee of the Azienda Ospedaliera Universitaria Senese in Italy, by the Ethics Committee of Hospital General Universitario Gregorio Marañón in Spain (VoTASD 391/20) and by the King’s College London Research Ethics Management in the UK (LRS-20/21-21196).

REDCap complies with the data protection regulations of the GDPR. The content of the data is encrypted by the platform itself. Survey respondents did not need to provide any information that could identify them (name, email, address, etc.). The collected data were shared with research collaborators involved in the study.

### Data analysis

Since the survey was administered electronically, data were downloaded and transferred for further analysis. Comprehensive descriptive analyses were performed using IBM SPSS Statistics for Windows, Version 25.0. [30]. Given the nature of the data, analyses performed consisted of using means, standard deviations and percentages to describe the sample.

## Results

### Sample characteristics

A total of 712 respondents initiated the survey, and 663 met the inclusion criteria stated above (see Tables 1 and 2).

**Table 1.** Sample characteristics

Sample characteristics	
Age of respondents in years, mean (SD)	44 (8.08)
Sex of person completing the survey (%male, % female)	16%, 84%
Autistic child’s age at the time of survey, mean (SD)	10 (4.39)
Sex of autistic child (%male, %female)	77%, 23%

**Table 2.** Autistic child’s gender per country of residency

Country	Male	Females	Total
Italy	129	29	158
Spain	222	65	287
UK	158	60	218
Total sample	509	154	663

### Early detection services

Parents or family members (70%) were usually the first to notice something different in a child’s development and/or behaviour, followed by school/nursery staff (19%), while 6% of public health professionals (e.g., nurses, paediatricians, family doctors/GPs) raised such concerns.

**Graph 1.**
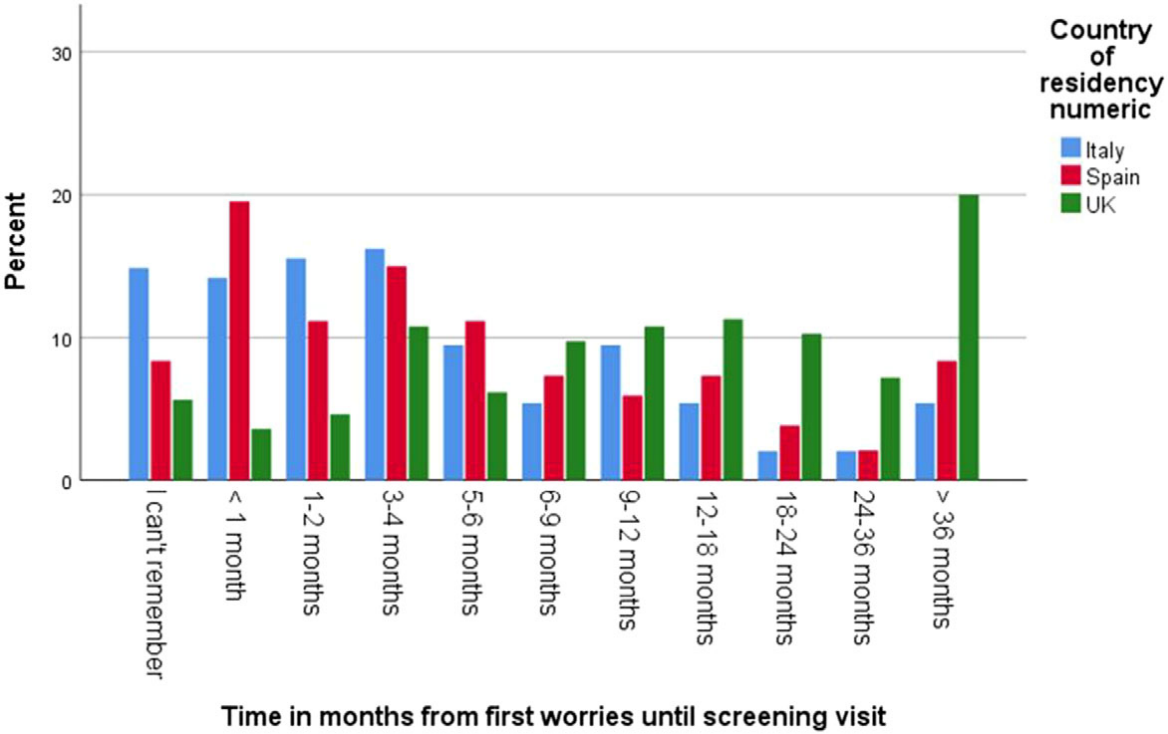
Time in months from first concerns until screening visit.

**Graph 2.**
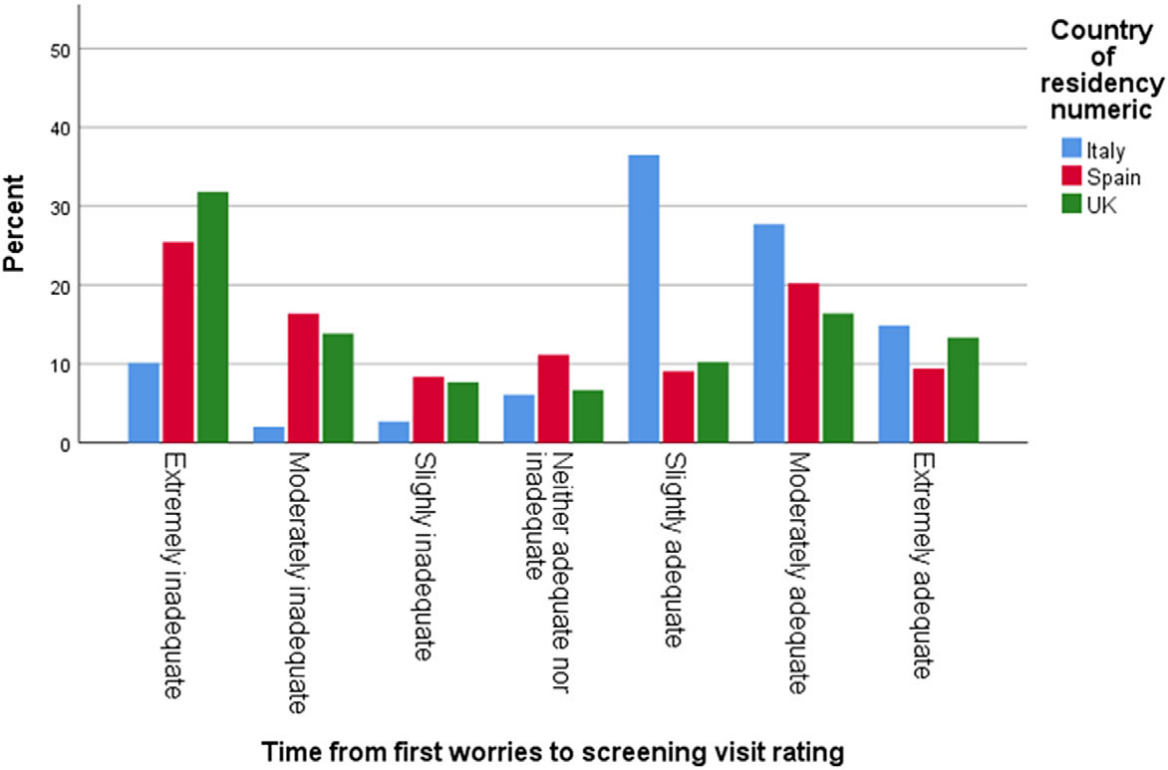
Rating of time from first concerns to screening visit.

The average age of a child when respondents started to have concerns was 12-18 months. Among the respondents, 46% in Italy, 44% in the UK, and 36% in Spain reported having received no guidance or support after raising their first concerns to their assigned professional. 49% of respondents in the UK, 22% in Spain, and 15% in Italy stated that it took over one year until a screening/detection appointment took place. A fifth of the UK sample (20%) reported that this process took over 36 months. 32% of respondents in the UK, 25% in Spain, and 10% in Italy rated these wait times as extremely inadequate (Graphs 1 and 2).

### Diagnostic services

**Graph 3.**
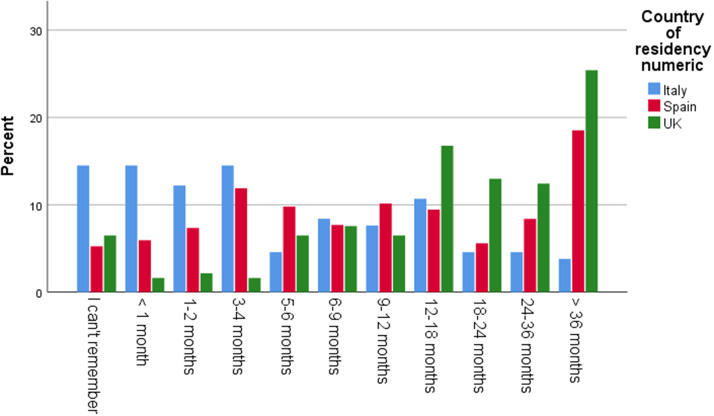
Time in months from screening visit until diagnosis confirmation.

**Graph 4.**
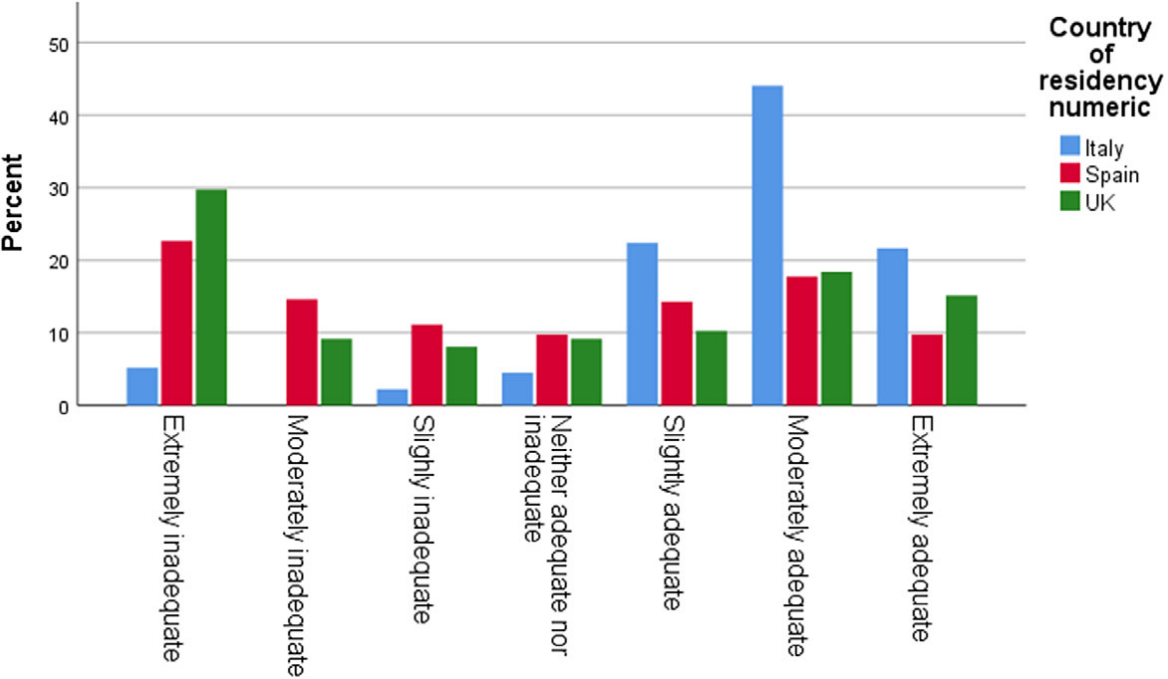
Rating of time from screening visit until diagnosis confirmation.

In this regard, 68% of respondents in the UK, 42% in Spain, and 24% in Italy reported that it took over one year from the screening visit to receive a confirmed diagnosis: 25% in the UK and 19% in Spain stated this process took over 36 months, while 30% of respondents in the UK and 23% in Spain rated these wait times as extremely inadequate (Graphs 3 and 4).

Despite these delays, 85% of respondents in Italy, 76% in the UK, and 55% in Spain stated that staff professionalism during diagnostic assessments was moderately to extremely adequate.

### Intervention

**Figure 1.**
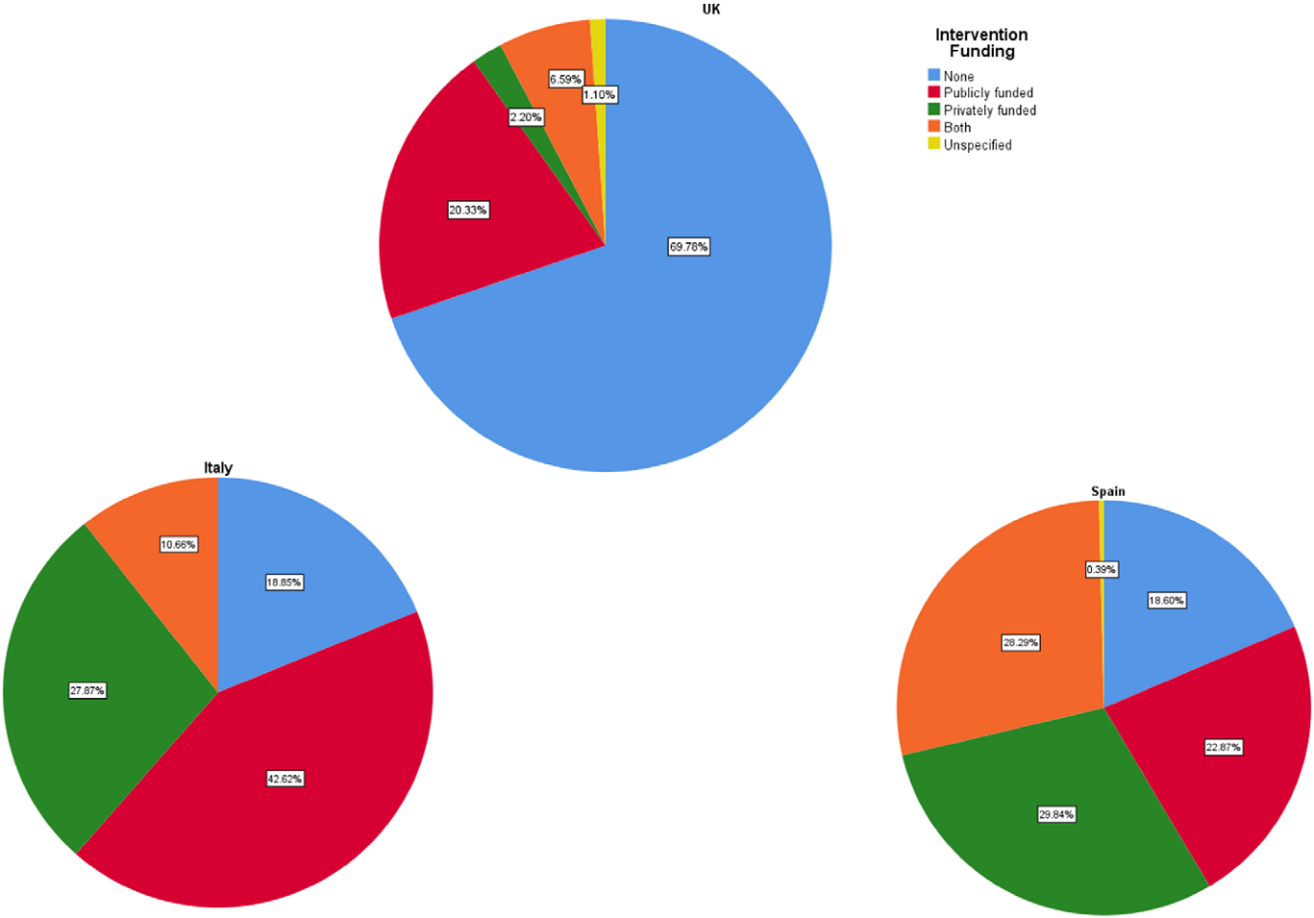
Type of intervention funding in Italy, Spain, and the UK.

In this regard, only 30% of respondents in the UK stated that the autistic children received any intervention after diagnosis, compared to 80% in Italy and 82% in Spain. However, only a small percentage in Italy and Spain of such interventions were publicly funded (Figure 1).

**Graph 5.**
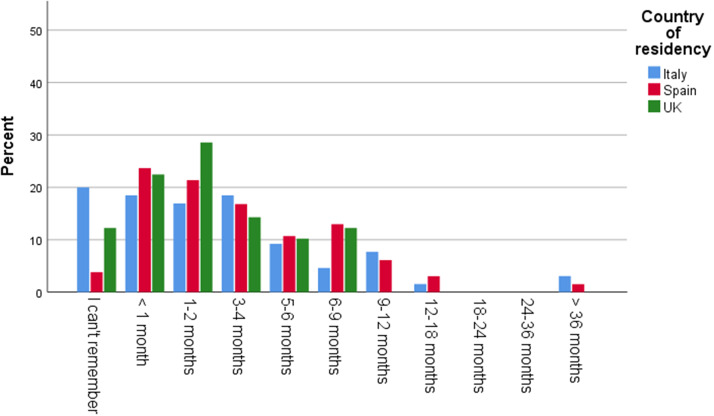
Time from diagnosis until publicly funded intervention in months.

**Graph 6.**
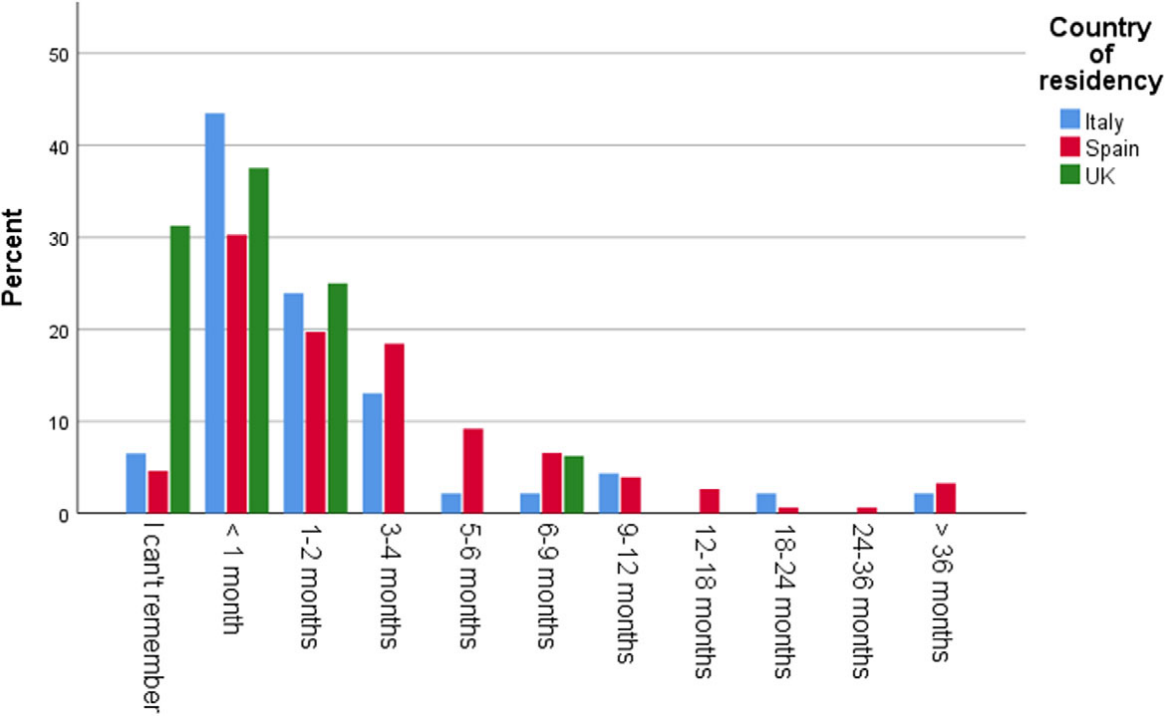
Time from diagnosis until privately funded intervention in months.

Only 24% of respondents in Spain, 22% in the UK, and 19% in Italy said the time from a confirmed diagnosis until a publicly funded intervention was less than one month, while 44% in Italy, 38% in the UK, and 30% in Spain stated that it took them less than one month to start a privately funded intervention (Graphs 5 and 6).

**Graph 7.**
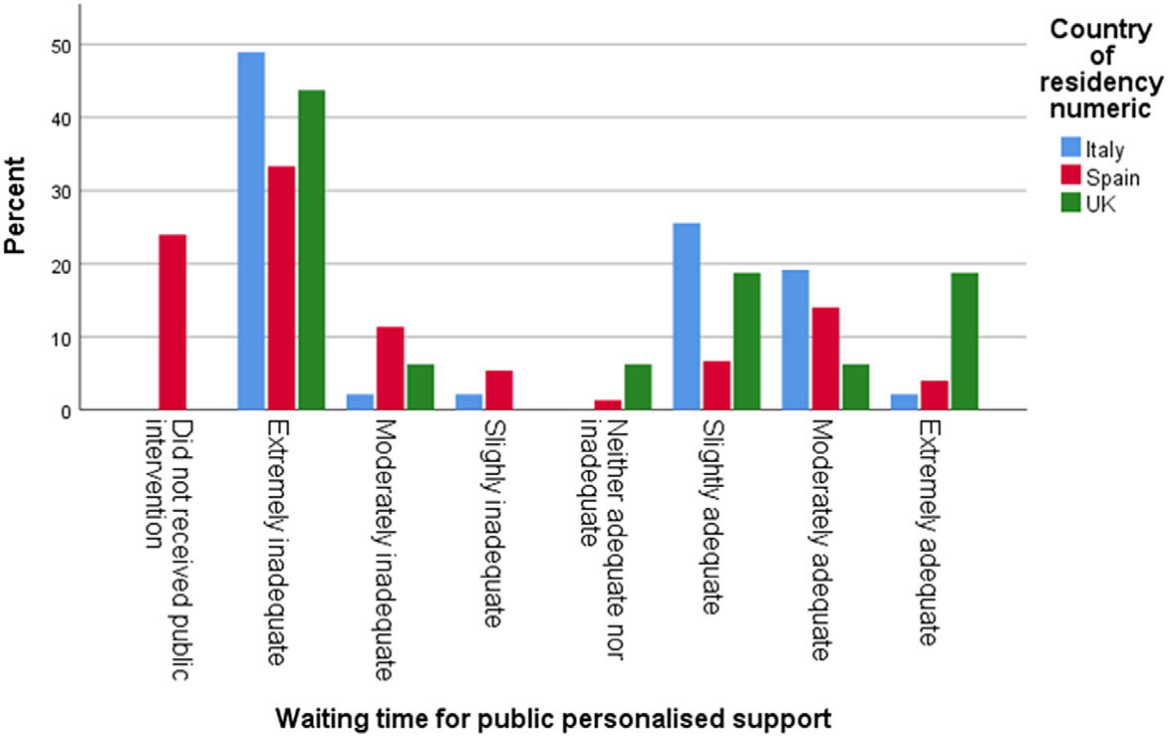
Waiting time for public intervention.

**Graph 8.**
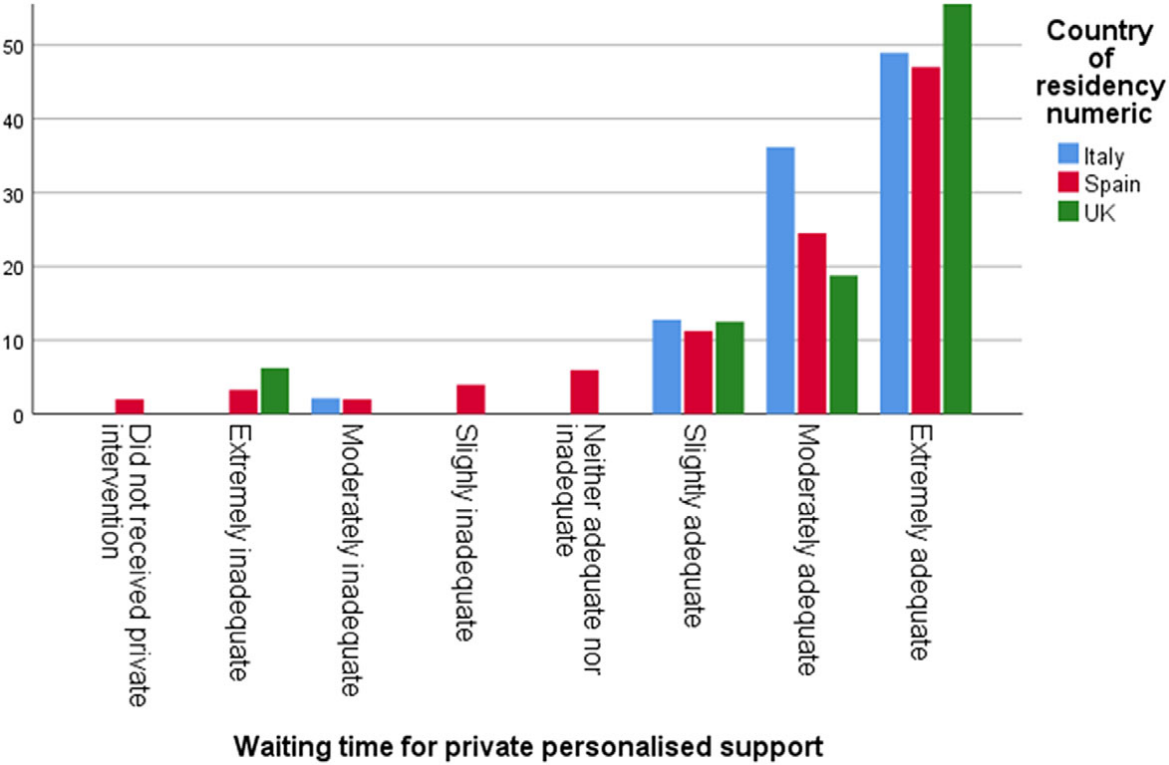
Waiting time for private intervention.

Furthermore, 45% of respondents in the UK and 30% in both Italy and Spain reported that the wait time from diagnosis until initiation of publicly funded therapeutic services was extremely inadequate, while 38% of respondents in Spain and 36% in Italy stated that the wait time from diagnosis until a privately funded intervention was extremely adequate (Graphs 7 and 8).

In addition, 47% of respondents in Italy stated that the information they had received about the intervention programme was moderately adequate, while 26% of the respondents in the UK reported that such information was extremely inadequate.

## Discussion

Our assessment of the autism care pathway identified several critical barriers to an optimal patient and carer journey in Italy, Spain, and the UK. Here we also discuss the possible underlying causes of these gaps.

### Information about autism and how to access early detection services

In our study, the average age when parents or family members reported concerns about a child’s development and/or behaviour was 12–18 months of age. This result is in keeping with the ASDEU study where the average age of first concerns was 18 months [22]. Added to this, once concerns were raised, a high percentage (62%) of respondents reported that it was not easy to access information about early detection services. This gap seems to be caused by lack of information and awareness about autism, early signs of autism, and services availability among the general population [20, 22, 31].

### Early detection/screening and diagnosis

The UK National Institute of Health and Care Excellence (NICE) provides evidence-based guidance on recognition, referral, and diagnostic assessment of autism in under 19s [23]. These guidelines state that referral to autism diagnostic services should occur if concerns regarding development or behaviour are raised by carers. These guidelines also recommend that if the screening visit finds that symptoms indicate autism, a diagnostic assessment should start within 3 months.

In this study, we found significant delays in autism-specific screening and subsequent diagnostic assessment. An alarmingly high proportion of respondents stated that it took them over a year from the time they raised their first concerns until they were offered a screening visit. Subsequently, 44% of respondents said it also took them over a year from that visit until they started a diagnostic assessment. These gaps in the care pathway may be caused by the insufficient availability of publicly funded autism specialist clinics and autism-trained specialists [25, 32, 33]. This leads to overwhelming of the available services, resulting in long waiting lists.

We found that parents or family members (70%) were usually the first to notice something different in a child’s development and/or behaviour, while only 6% of public health professionals raised such concerns. The latter may be due to a lack of triaging programmes at well-baby clinics and a lack of autism-trained health and educational professionals able to identify early signs of autism. Early detection should not mainly rely on the carers.

Our results are in keeping with some of the ASDEU study findings. In this study, 70% of respondents stated that the first person to notice and report something different in a child’s development was a parent or family member. It also found that most families reported a delay of over 6 months in accessing detection and diagnosis services [22].

Similarly, the Autism Speaks Global Autism Public Health Initiative (GAPH), reported that the age of first concerns was 24.4 months, and the majority of respondents stated that it was also a family member who raised first concerns. Furthermore, 35% of respondents reported difficulties or delays accessing services: 29% reported that this was due to long waiting lists, and 31% said this was due to lack of information.

### Early intervention

Research shows that earlier intervention in autism is more likely to have a major long-term positive effect on symptoms and may improve prognosis in a significant proportion of autistic children [33, 34]. NICE (2011) recommends that, after diagnosis, a case manager from the autism specialist team be available to coordinate support and treatment and to reassess needs through childhood and adolescence.

As in the ASDEU study [22], we observed lacks and delays in the initiation of therapeutic intervention. Only 30% of respondents in the UK stated that the autistic children received intervention after diagnosis, whereas 80% in Italy and 82% in Spain reported that they did. However, a good proportion of these relied on private funding or a combination of private and public funding.

Added to this, we found that the wait times between confirmed diagnosis and publicly funded intervention were less than 3 months in only 51 % of respondents in the UK, 45% in Spain, and 35% in Italy. Not surprisingly, times were shorter when there was private funding. Similarly to our findings, the ASDEU study found that most respondents reported delays of 0–3 months to access intervention services after confirmation of diagnosis [22]. They found that delays between detection and diagnosis were longer than delays between diagnosis and intervention. These findings suggest that delays in screening and diagnosis may be a cause of delays in intervention.

Our results highlight the fact that current publicly funded therapeutic services are unable to accommodate the number of autistic children in need of these services nor the needs of a growing autistic population. This results in long waiting lists or the necessity to rely on privately funded services. Perhaps this is also due to a lack of appropriately trained staff to administer these therapies.

In addition to this treatment gap, another significant barrier to providing high-quality post-diagnostic support is a lack of evidence for the effectiveness and cost-effectiveness of pharmacological and psychological/psychosocial interventions [25, 35, 36]. This is concerning, given the lifelong nature of autism and therefore the need for long-term personalised care adapted to the needs of autistic children as they become adults.

### Support for carers of autistic children

NICE recommends that, once the diagnosis is confirmed, families be provided with contact details for local and national support organisations, as well as organisations that can provide advice on access to welfare benefits, educational support, and social care [27].

In our study, we observed an overall lack of support for carers. 41% of respondents reported receiving no guidance or support after raising their first concerns to their assigned professional. Furthermore, 30% of respondents said they received very little or no support after the diagnosis was confirmed. Our results are similar to what has been reported in the UK previously, with only 4% of families reporting being fully supported in the 12 months following diagnosis, with many relying on self-research to be able to understand what the diagnosis means for them and what support they need and are entitled to [32].

In addition, 58% of respondents said they had not received any training, coaching, or counselling to help them cope with their child’s difficulties. Most families of autistic children want and need more guidance, counselling, and emotional support to help them understand the meaning and the implications of the diagnosis in order to be able to support their autistic children, avoid crisis in the family, and manage stress adequately [12, 25].

Even though there are some clear recommendations regarding personalised care pathways for early identification, referral, and assessment of autism, these recommendations are not consistently adhered to due to wide local and regional variations in resource availability, and wait times for intervention can be significant [25, 32, 33]. Efforts to improve the care pathway are paramount to ensuring the best outcomes for autistic people, their families, the community, and society – particularly given that autism research receives far less funding than other conditions with comparable prevalence and/or human economic impact [37].

The findings of this study need to be understood in the context of a number of limitations. First, we cannot estimate the rate of response for this survey as it is not possible to know the number of people who received an invitation to complete it and the number of people who were reached by our social networks. Secondly, the nature of our sample needs to be taken into consideration. It is crucial to note that the survey results were derived from a convenience sample and that the survey was restricted to people with internet access, active in social media, receiving services in the sites involved, or in contact with local associations, which may not be representative of all service users. Furthermore, our sample was highly educated (most respondents had a college degree or higher).

Unfortunately, the quality of both the diagnostic processes and the interventions varies across different countries, cities, and rural areas, so a lack of information on the size of the respondents’ area of residence and the services available there is a shortcoming of the study. Future studies should make extra efforts to recruit populations of diverse socio-economic and educational backgrounds to ensure better representation and more generally to capture their difficulties.

In addition, since this study is comprehensive in its focus on the full care pathway from initial screening to intervention, our sample is somewhat biased by parent respondents who have actively been seeking, or in touch with, support services. In contrast, our survey does not well capture cases whereby diagnosis was never made after initial concerns and/ or screening – for example, due to the individual not meeting threshold on standardised measures, or due to family disengagement with the diagnostic process. Ease of access of relevant support for those who do not receive a diagnosis – some of whom may be from underrepresented populations where standardised screening tools are known to underestimate the autism phenotype (e.g., for autistic girls and those with co-occurring intellectual disability) – as well as reasons for family disengagement (which may, in some cases, result from dissatisfaction with services) would therefore be important to investigate further, as these issues may complement our own findings on areas where the autism care pathway requires optimisation.

In the meantime, we believe findings from our study shed light on current pathway issues and will help inform future studies and policy harmonisation in Europe (Box 1) to improve the journey of autistic people and their carers, and their overall quality of life.

Box 1. Policy recommendations.

- Raise awareness among parents, family members, the community, and primary care providers about developmental milestones, the early signs of autism, and the importance of early detection and intervention.
- Encourage professionals to listen to family concerns regarding delays in milestones and atypical neurodevelopmental signs and address them immediately. Parents/carers have proven to be good informants for raising these concerns and recognising warning signs suggestive of autism.
- Increase autism-specific triage programmes at well-baby clinics.
- Provide autism training to first-line health services professionals such as health visitors, nurses, family doctors, GPs, and paediatricians.
- Reduce the delay in screening and diagnosis, thus enabling children to begin intervention programmes earlier.
- Increase support to families of children diagnosed with autism.
- Perform further assessment in order to identify and learn from countries/areas with better patient journey experiences.
- Reduce economic inequality.

## Supporting information

Mendez et al. supplementary material Mendez et al. supplementary material

Mendez et al. supplementary material Mendez et al. supplementary material

## Supplementary Material

The supplementary material for this article can be found at http://doi.org/10.1192/j.eurpsy.2023.2435.
