# Supplementary material for: Autism care pathway in Europe
Source: Eur Psychiatry. 2023 Sep 11;66(1):e81. doi: 10.1192/j.eurpsy.2023.2435 (PMC10594203; doi:10.1192/j.eurpsy.2023.2435)
Supplement: Mendez et al. supplementary material [file S0924933823024355sup001.docx]

**Supplementary material.**

**Appendix 1: Survey in English.**

**BACKGROUND INFORMATION:**

**These questions should be answered by the parent/carer of the person on the autism spectrum.**

The questions in this initial section will ask you about your sex, where you live, your age, your education, the current monthly income of the household where the child with autism lives and the number of people living in the home.

1. Please state your country of residence
2. County
3. Town/City
4. Is one of the parents a migrant? Yes/No. Country of origin:
5. Your biological sex (sex assigned at birth)

Male ⭘

Female ⭘

1. How old are you?
2. Who is answering this questionnaire?

I am a parent of a person on the autism spectrum ⭘

I am a person on the autism spectrum and a parent of a person on the autism spectrum ⭘

I am a carer of a person on the autism spectrum (not a blood relative) ⭘

I am a blood relative of a person on the autism spectrum (not a parent).

Sibling ⭘

Other ⭘

Please specify:

1. Approximately how many people live in your city/town?

Fewer than 10,000 ⭘

Between 10,000 and 50,000 ⭘

Between 50,000 and 150,000 ⭘

Between 150,000 and 1,000,000 ⭘

More than 1,000,000 ⭘

1. Please indicate the highest level of education completed in your household (by you or your partner).

No formal education completed ⭘

Primary education (or similar: elementary, middle school, etc.) ⭘

Secondary School (high school) ⭘

Further education (College) ⭘

Undergraduate degree (Bachelor’s) ⭘

Postgraduate Education (Master’s, PhD, etc.) ⭘

1. How many people live in the same household as the child with autism?
2. Optional question: Please state your approximate current monthly household income (in your country’s currency).

< 500 € ⭘

Between 500-1000€ ⭘

1000-1500€ ⭘

1500-2000€ ⭘

>2000€ ⭘

Without income ⭘

I would rather no answer ⭘

1. What is the age of the child with autism?
2. What is the biological sex (assigned at birth) of the child with autism?

Male ⭘

Female ⭘

1. What is the verbal ability of the child with autism?

Does not talk ⭘

Uses single words only (e.g. “daddy”, “mommy”) ⭘

Uses two or three word phrases (e.g. “want biscuit”) ⭘

Uses sentences with four or more words (e.g. “I want a biscuit”) ⭘

Uses complex sentences (e.g. “When we get home, can I have a biscuit?”) ⭘

**DETECTION/SCREENING:**

**These questions should be answered by the parent/carer of the person on the autism spectrum.** In this section, you will answer questions about the process of detecting the child’s difficulties (or your difficulties if you are the person on the autism spectrum) before receiving the diagnosis.

1. How old was the child (or you if you are the person on the autism spectrum) when you or someone else first noticed developmental issues?

I can’t remember ⭘

Less than 1month ⭘

From 1 to 2 months ⭘

From 3 to 4 months ⭘

From 5 to 6 months ⭘

From 6 to 9 months ⭘

From 9 to 12 months ⭘

From 12 to 18 months ⭘

From 18 to 24 months ⭘

From 24 to 36 months ⭘

Over 36 months ⭘

1. Who was the first person who suspected that something was different about the child’s development?

You ⭘

A family member ⭘

Please specify____________________________

Paediatrician or nurse from public health service ⭘

Paediatrician or nurse from private health services ⭘

A teacher or staff from nursery, preschool, or school ⭘

Other ⭘

Please specify____________________________

1. Would you say it was easy to access information about programmes and early detection services in your area?

Yes ⭘

No ⭘

Please explain why_____________________

1. What was the next step in the detection process?

We had to look for a diagnostic service ourselves ⭘

We received a letter with a medical appointment from the hospital ⭘

The professional who had the first concern referred us directly to a specialized service ⭘

Other ⭘

Please specify ______________________________________

1. How many months went by from first concerns until there was an assessment by a GP/family Dr or paediatrician?

I can’t remember ⭘

Less than 1month ⭘

From 1 to 2 months ⭘

From 3 to 4 months ⭘

From 5 to 6 months ⭘

From 6 to 9 months ⭘

From 9 to 12 months ⭘

Over 12 months ⭘

Over 18 months ⭘

Over 24 months ⭘

Over 36 months ⭘

1. Did you have any professional guidance and support to address your first concerns?

Please check all that apply:

No, I did not received any support/guidance ⭘

Paediatrician ⭘

Psychologist ⭘

Psychiatrist ⭘

Nurse ⭘

Child neurologist ⭘

Preschool/school teacher ⭘

Other ⭘

Please specify: __________________________

1. If the detection/screening process was privately funded, please tell us how much it cost you in total?
2. How adequate do you consider the detection process?

|  | Extremely adequate | Moderately  adequate | Slightly adequate | Neither adequate nor inadequate | Slightly inadequate | Moderately inadequate | Extremely inadequate |
| --- | --- | --- | --- | --- | --- | --- | --- |
| The time it took from the first suspicion of developmental problems until the initial screening/detection visit |  |  |  |  |  |  |  |
| The qualifications of the staff who worked with the child during the detection process |  |  |  |  |  |  |  |
| The degree to which the professionals involved in the process listened and addressed your concerns |  |  |  |  |  |  |  |
| Overall detection process |  |  |  |  |  |  |  |

1. In the screening/early detection phase, to what extent did the professionals who worked with the child …….

|  | A lot | Somewhat | Moderately | Slightly | Very little | Not at all |
| --- | --- | --- | --- | --- | --- | --- |
| Take enough time to talk to you about the child’s difficulties? |  |  |  |  |  |  |
| Offer support? |  |  |  |  |  |  |
| Give you an opportunity to make decisions about using different tests? |  |  |  |  |  |  |
| Inform you about the results of the evaluation? |  |  |  |  |  |  |

1. If you have any suggestions about the process for detecting early autism signs, please enter below.

**DIAGNOSIS:**

**These questions should be answered by the parent/carer of the person on the autism spectrum.** In this section, you will be asked about the diagnostic process. We are interested in your perception of the direct care you have been receiving at the different centres or institutions that have cared for the child during the process. The questions in this section refer to all professionals and institutions that have been directly involved in the diagnostic process.

1. Has the child received any of the following diagnoses?

Autism spectrum disorder (ASD) ⭘

Autistic disorder/childhood autism ⭘

Asperger’s syndrome/ Asperger’s disorder ⭘

Atypical autism ⭘

Pervasive developmental disorder (PDD) ⭘

Pervasive developmental disorder - not otherwise specified (PDD-NOS) ⭘

Other ⭘

Please specify______________________________________________

1. At what age was the child diagnosed with autism spectrum disorder?
2. Do you recall how much time went by from first concerns or the primary care assessment (detection/screening visit) until the diagnosis was confirmed by an autism specialist?

I can’t remember ⭘

Less than 1month ⭘

From 1 to 2 months ⭘

From 3 to 4 months ⭘

From 5 to 6 months ⭘

From 6 to 9 months ⭘

From 9 to 12 months ⭘

Over 12 months ⭘

Over 18 months ⭘

Over 24 months ⭘

Over 36 months ⭘

1. Which professionals assisted you in the diagnostic process? Please tick all that apply (multiple answers are possible).

No, I did not received any support ⭘

Nurse ⭘

Family doctor (GP) ⭘

Paediatrician ⭘

Child neurologist ⭘

Psychologist ⭘

Psychiatrist ⭘

Other ⭘

Please specify _____________

1. Please specify any other conditions ever diagnosed by a doctor of the person on the autism spectrum. Please tick all that apply (multiple answers are possible).

Don’t know ⭘

None ⭘

Epilepsy ⭘

Anxiety disorder ⭘

Depression ⭘

Schizophrenia/Psychosis ⭘

Bipolar disorder ⭘

Attention deficit hyperactivity disorder (ADHD) ⭘

Obsessive compulsive disorder ⭘

Personality disorder ⭘

Behavioural disorder ⭘

Tics/Tourette's syndrome ⭘

Down syndrome ⭘

Intellectual disability ⭘

Learning disorder, e.g. dyslexia ⭘

Sleep disturbance ⭘

Gastrointestinal disorder ⭘

Other ⭘

Please specify: ____________________________________

If the diagnostic assessment was privately funded, please tell us how much did this cost you in total?

1. Do you recall if any of these tests were done? Please tick all that apply (multiple answers are possible).

ADOS ⭘

ADOS-2 ⭘

ADI-R ⭘

IQ test ⭘

Can’t remember ⭘

Other ⭘

Please specify____________________________________

1. If privately funded, how much did ADOS cost you?
2. If privately funded, how much did ADOS-2 cost you?
3. If privately funded, how much did ADI-R cost you?
4. If privately funded, how much did IQ test cost you?

If privately funded, how much did you pay for the above mentioned tests?

What score did the child obtain on the most recent intelligence (IQ) test?

________________

Don’t know ⭘

Prefer not to answer ⭘

Has the child ever had an electroencephalogram (EEG)?

Yes ⭘

No ⭘

<If the answer is No please go to question 42>

If yes, at what age?

Was EEG public or privately funded?

Public ⭘

Private ⭘

If privately funded, how much did EEG cost you?

1. Did you receive advice or information from the professionals who gave you the autism spectrum diagnosis report? Please tick the box for the areas where you received appropriate or enough information (multiple answers are possible).

No ⭘

Medical needs (specialist, medicine, genetic counselling) ⭘

Educational needs (centres, support) ⭘

Social needs (organizations, family support) ⭘

Materials (bibliography, agencies, web pages, etc.) ⭘

Other ⭘

Please specify:_____________________________________

1. How adequate do you consider the diagnostic process?

|  | Extremely adequate | Moderately adequate | Slightly adequate | Neither adequate nor inadequate | Slightly inadequate | Moderately inadequate | Extremely inadequate |
| --- | --- | --- | --- | --- | --- | --- | --- |
|  |  |  |  |  |  |  |  |
| The time it took from the screening/detection visit until diagnosis confirmation |  |  |  |  |  |  |  |
| The professionalism of the staff who handled the diagnostic process |  |  |  |  |  |  |  |
| The information and support you received from these professionals |  |  |  |  |  |  |  |
| The overall diagnostic process |  |  |  |  |  |  |  |

1. To what extent did the professionals who worked with the child on the autism spectrum……

|  | A lot | Somewhat | Moderately | Slightly | Very little | Not at all |
| --- | --- | --- | --- | --- | --- | --- |
| Take enough time to talk to you about your difficulties? |  |  |  |  |  |  |
| Offer support? |  |  |  |  |  |  |
| Give you an opportunity to make decisions about using different tests? |  |  |  |  |  |  |
| Inform you about the results of the assessment? |  |  |  |  |  |  |
| Give you information about the services offered by the institutions or other services in your community? |  |  |  |  |  |  |
| Give you advice on how to access more information |  |  |  |  |  |  |
| Give you advice on how to contact other parents or people in the same situation (e.g. parent organisations or specialised centres?) |  |  |  |  |  |  |
| Coordinate things so that all services involved provided information to arrive at a coherent diagnosis? |  |  |  |  |  |  |

1. If you have any suggestions for the diagnostic programmes, please enter below.

**EARLY INTERVENTION:**

**These questions should be answered by the parent/carer of the person on the autism spectrum.** In this section of the survey, you will be asked about the type and quantity of early intervention services that the child received soon after diagnosis.

1. Did the child receive any support or treatment soon after diagnosis?

Yes ⭘

No ⭘

<If the answer is NO please skip to question 56>

1. Do you remember how much time went by after the diagnosis was confirmed until the intervention programme started?

I can’t remember ⭘

Less than 1month ⭘

From 1 to 2 months ⭘

From 3 to 4 months ⭘

From 5 to 6 months ⭘

From 6 to 9 months ⭘

From 9 to 12 months ⭘

From 12 to 18 months ⭘

From 18 to 24 months ⭘

From 24 to 36 months ⭘

Over 36 months ⭘

1. What types of intervention, both privately and publicly funded, did the child receive?

Publicly funded ⭘

Please specify: _________________________

Privately funded ⭘

Please specify: _________________________

1. How many publicly funded intervention sessions did the child receive on a weekly basis?

<If the answer is none please skip to question 51>

1. Please indicate how long each session lasted on average.

30 minutes ⭘

1 hour ⭘

1-2 hours ⭘

Over 2 hours ⭘

3 months

3-6 months

6-12 months

over 12 months

1. How many privately funded intervention sessions did the child receive on a weekly basis?

<If the answer is none please skip to question 54>

1. Please indicate how long each session lasted on average.

30 minutes ⭘

1 hour ⭘

1-2 hours ⭘

Over 2 hours ⭘

3 months

3-6 months

6-12 months

over 12 months

1. If the interventions were privately funded, please tell us how much each of these sessions costed you?
2. How were the intervention sessions conducted? Please tick all that apply (multiple answers are possible).

In group ⭘

Individual ⭘

Other ⭘

Please specify:__________________________________

1. How far from your home was the facility where regular interventions took place?

30 minutes ⭘

1 hour ⭘

1-2 hours ⭘

Over 2 hours ⭘

1. Has the child ever received …

| Treatment/Intervention | yes | no |
| --- | --- | --- |
| **Behavioural treatment**, e.g. Applied Behaviour Analysis (ABA), Pivotal Response Training (PRT), Lovaas, Discrete Trial Training (DTT)? |  |  |
| **Developmental treatment**, e.g. Relationship Developmental Intervention (RDI), Early Start Denver Model (ESDM)? |  |  |
| Relationship-based treatment, e.g. Developmental, Individual Difference, Relationship-based (DIR) Model/Floortime, Therapie d’echange et de developpement (TED)? |  |  |
| TEACCH programme? |  |  |
| Portage intervention? |  |  |
| Psychoanalytic treatment? |  |  |
| Speech and language therapy? |  |  |
| Occupational therapy/physiotherapy? |  |  |
| Parent training/coaching/counselling to help you with the child? |  |  |
| Other psychological/educational/behavioural treatment? |  |  |

1. How adequate do you consider the intervention process in regards to the following?

|  | Extremely adequate | Moderately adequate | Slightly adequate | Neither adequate nor inadequate | Slightly inadequate | Moderately inadequate | Extremely inadequate |
| --- | --- | --- | --- | --- | --- | --- | --- |
| The waiting time to receive a public intervention programme |  |  |  |  |  |  |  |
| The waiting time to receive a  private intervention programme |  |  |  |  |  |  |  |
| The number of sessions that the child received |  |  |  |  |  |  |  |
| The information that you received about the programme |  |  |  |  |  |  |  |

1. If you have any suggestions for early intervention programmes, please enter below.

**EPILEPSY-SPECIFIC QUESTIONS:**

These questions need to be answered only if the child or you have experienced seizures/fits or have been diagnosed with epilepsy.

1. Has the child been diagnosed with epilepsy?

Yes ⭘

No ⭘

<If your answer is No please skip to question 67>

1. At what age was the child diagnosed with epilepsy?
2. Is the child on medication to treat epilepsy?
3. At what age was the child prescribed medication for the treatment of epilepsy?
4. Who prescribed this medication?

Psychiatrist ⭘

Child neurologist ⭘

Neurologist ⭘

Other ⭘

Please specify: _____________

1. If this was privately funded, how much does each appointment cost you?
2. How often do you need to see this doctor?
3. If on medication, please complete this table:

| Name of drug | Dosage (if known) | Dose frequency | Paid for directly by carer (Yes/No) | If yes, how much does it cost per month? (£) |
| --- | --- | --- | --- | --- |
|  |  |  |  |  |
|  |  |  |  |  |
|  |  |  |  |  |

**COVID-19 SERVICE-RELATED ACCESS QUESTIONS:**

1. Have you attempted to access services related to COVID-19?

Yes ⭘

No ⭘

<If the answer is No please skip to question 71>

1. What type of service was this? Select all that apply from the high level list of service categories below (multiple answers are possible).

Mental health crisis support ⭘

Emergency telephone medical service, e.g. national medical hotline ⭘

Accident and emergency medical services ⭘

Intensive care unit ⭘

Other (please specify): ________________________________________________

1. Did you experience any difficulty accessing any of them? Please tick all that apply (multiple answers are possible).

None ⭘

Mental health crisis support ⭘

Emergency telephone medical service, e.g. national medical hotline ⭘

Accident and emergency medical services ⭘

Intensive care unit ⭘

Other (please specify): ________________________________________________

1. Have you experienced discrimination in accessing any of these services because the child has ASD?

No ⭘

Yes ⭘

Please describe: ________________________________________________

**FINAL QUESTION**

1. Is there something else you want to tell us that we didn’t cover during this survey?

|  |
| --- |

End of questionnaire

Thank you for your participation!
